# Structure and Phase State of Ti_49.4_Ni_50.6_ (at%) Hydrogenated in Normal Saline

**DOI:** 10.3390/ma14227046

**Published:** 2021-11-20

**Authors:** Victor Grishkov, Aleksandr Lotkov, Dorzhima Zhapova, Yuri Mironov, Victor Timkin, Elena Barmina, Olga Kashina

**Affiliations:** Institute of Strength Physics and Materials Science, Siberian Branch of the Russian Academy of Science, 634055 Tomsk, Russia; grish@ispms.ru (V.G.); lotkov@ispms.ru (A.L.); myp@ispms.ru (Y.M.); timk@ispms.tsc.ru (V.T.); barmina@ispms.tsc.ru (E.B.); ocash@ispms.tsc.ru (O.K.)

**Keywords:** Ti_49.4_Ni_50.6_ (at%) alloy, hydrogen, electrolytic hydrogenation, hydride structure

## Abstract

The paper analyzes the surface structure and phase state of Ti_49.4_Ni_50.6_ (at%) hydrogenated at 295 K in normal saline (0.9% NaCl aqueous solution with pH = 5.7) at 20 A/m^2^ for 0.5–6 h. The analysis shows that the average hydrogen concentration in the alloy increases with the hydrogenation time t_H_ as follows: slowly to 50 ppm at t_H_ = 0.5–1.5 h, steeply to 150 ppm at t_H_ = 1.2–2 h, and linearly to 300 ppm at t_H_ = 2–6 h. According to Bragg–Brentano X-ray diffraction data (θ–2 θ, 2 θ ≤ 50°, CoKα radiation), the alloy in its scanned surface layer of thickness ~5.6 µm reveals a TiNiH_x_ phase with x = 0.64 and x = 0.54 after hydrogenation for 4 and 6 h, respectively. The structure of this phase is identifiable as an orthorhombic hydride similar to β_1_–TiFeH_0.94_ (space group Pmcm), rather than as a tetragonal TiNiH_x_ hydride with x = 0.30–1.0 (space group I4/mmm). Time curves are presented to trace the lattice parameters and volume change during the formation of such an orthorhombic phase from the initial cubic B2 phase in Ti_49.4_Ni_50.6_ (at%).

## 1. Introduction

TiNi alloys, showing superelasticity and shape memory, good plasticity, high corrosion resistance, and biocompatibility, are efficient materials for manufacturing various engineering devices [1,2] and medical implants [3,4]. At the same time, such alloys after their long contact with a hydrogen-containing environment, e.g., biological, are prone to embrittlement [5,6,7,8]. At near-room temperatures (290–310 K), the rate of diffusion process in TiNi is low such that a large amount of hydrogen first goes into its surface layers and then diffuses deep into the material [9,10]. As has been shown [10,11,12], the saturation of TiNi alloys with hydrogen decreases the temperatures of thermoelastic martensite transformations from a cubic B2 phase to a rhombohedral R and a monoclinic B19′ phase, and this impairs their superelasticity and shape memory effect [8,10,13,14] and causes their cracking and fracture [15,16]. One of the contributory factors for such functional and mechanical degradation is the formation of hydrides [16], and structural studies are needed to clarify their effect on the properties of TiNi alloys. Note that the data currently available on the state diagram of Ti-Ni-H system and the structures of hydride phases are rather scanty and contradictory.

The most comprehensive studies of such hydrides concern TiNiH_1.0_ formed via saturation in gaseous hydrogen [17,18]. The studies show that this type of hydride has a tetragonal structure (space group I4/mmm) with orientation relations [100]_H_ ‖ [100]_B2_, [010]_H_ ‖ [100]_B2_, [001]_H_ ‖ [001]_B2_ and lattice parameters *a*_H_ = *b*_H_ ≈ 2 *a*_B2_ and *c*_H_ ≈ 4 *a*_B2_ [18,19]. The lattice parameters of TiNiH_1.0_, according to [17,18], measure *a*_H_ = 6.221 (1) Å, *c*_H_ = 12.363 (3) Å [17] and *a*_H_ = 6.2165 (6) Å, *c*_H_ = 12.326 (1) Å [18]. From electron microscopy data [19], it follows that the hydride formed in Ti–48Ni–2Al (at%) via electrolytic hydrogenation in 4% H_2_SO_4_ water solution has the same tetragonal structure and space group I4/mmm as TiNiH in TiNi after gaseous hydrogenation [17,18]. The same phase appears after electrolytic hydrogenation in NaOH water solutions: TiNiH_x_ with x = 0.95) [20] and TiNiH_x_ with x = 0.30 and 0.85 [21]. According to [15,21,22], electrolytic hydrogenation in both NaOH and H_2_SO_4_ water solutions results in a tetragonal TiNiH_x_ phase whose lattice parameters depend on the hydrogen content. As the atomic ratio x varies from 0.30 to 0.55, the lattice parameter *a*_H_ changes from 6.09 Å to 6.25 Å and *c*_H_ from 11.40 Å to 12.60 Å. Tetragonal TiNiH_x_ phases with x from 0.30 to 0.32 are found in Ti–50.8Ni (at%) thin wires of diameter 0.4 mm immersed in hot (90 °C) H_3_PO_4_ [23].

Several papers analyze the state of binary TiNi and Fe-doped TiNi after gaseous hydrogenation [24] and electrolytic hydrogenation in water solutions with 4% and 5% H_2_SO_4_ [25,26,27]. As has been found [24,25,26], no tetragonal hydride TiNiH_x_ appears even at a hydrogen content in surface layers of up to 4500 ppm [24]. Instead, hydrogen-induced martensite corresponding to a monoclinic B19′ phase (space group P2_1_/m) is formed during hydrogenation [24,25,26,27]. Such a hydrogen martensite phase appears during hydrogenation at room temperature and even in those initially B2-structured alloys which escape transformations to B19′ on cooling up to 77 K without hydrogenation. Unfortunately, no discussion of factors that may provide the absence of tetragonal TiNiH_x_ at a high hydrogen content is presented in the cited papers [24,25,26,27].

Most of the studies of TiNi alloys hydrogenated in normal saline solutions (0.9% NaCl), e.g., [9,28,29,30,31], mainly concern the influence of hydrogenation parameters on hydrogen absorption, the effect of hydrogen on the mechanical properties of TiNi alloys (embrittlement), and the mechanisms of its thermal desorption. The structure of hydride phases is analyzed qualitatively by comparing the form of diffraction patterns after hydrogenation [28,29,30] with related data [17,18]. The lattice parameters of hydride phases in the majority of papers are not given. However, it is noted that certain reflections resulting from hydrogenation cannot be identified with a tetragonal hydride phase alone [9,16,23]. Besides, the presence of TiH_2_ in surface layers after hydrogenation in normal saline is reported but without any confirmation [31]. Thus, the studies of hydride structures formed during hydrogenation of TiNi based alloys must be continued now.

Here we analyze the effect of electrolytic hydrogenation in normal saline (0.9% NaCl aqueous solution with pH = 5.7) on the structure and phase state of Ti_49.4_Ni_50.6_ (at%).

## 2. Materials and Methods

**Figure 1:**
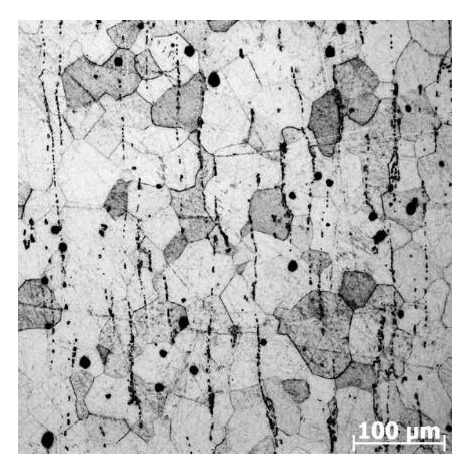
The optical microscopy image of microstructure in Ti_49.4_Ni_50.6_ (at%) samples in rolling plane.

**Figure 2:**
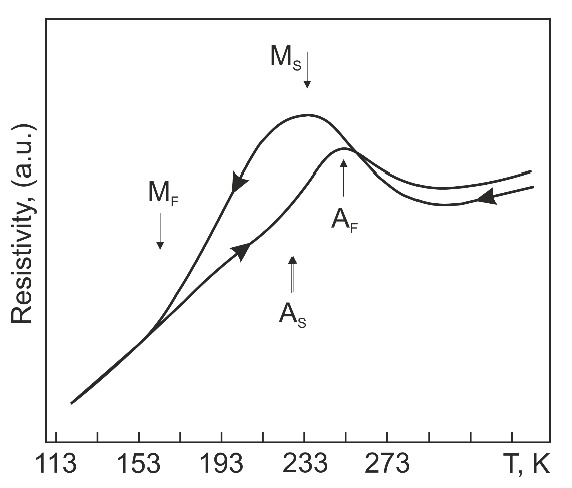
Temperature dependences of electrical resistivity on cooling and heating of initial samples.

The alloy under study was Ti_49.4_Ni_50.6_ (at%) supplied as rolled plates of thickness 1.2 mm by MATEK-SMA Ltd. (Moscow, Russia). The Axiovert-200M optical microscopy (Carl Zeiss AG, Oberkochen, Germany) was used for the study of alloy microstructure. In its as-received state, the alloy had a coarse-grained structure. The shape of grains in the rolling plane was quasi-equiaxed with an average aspect ratio of 1.4, Figure 1. The average grain size in this plane was 42 µm in the rolling direction and 29 µm crosswise, and its value perpendicular to the rolling plane was 14 µm. The martensitic transformation temperatures were analyzed from temperature dependences of sample resistivity measured by four-probe potential method. The rate on cooling and heating was 3 K/min. The liquid nitrogen was used at temperatures below 300 K. The temperature dependences of sample resistivity on cooling and heating are presented in Figure 2. On cooling and heating, the alloy undergoes B2↔B19′ martensitic transformations (MT), where B2 is a CsCl-ordered cubic phase and B19′ is a monoclinic martensitic phase. The start (M_S_) and finish (M_F_) temperatures of B2→B19′ MT are 234 K and 163 K, respectively. The temperature range of B19′→B2 MT is from 227 K (A_S_) to 252 K (A_F_). Thus, at room temperature (near 295 K), the alloy had a B2 structure. It must be noted that the Ti_49.4_Ni_50.6_ (at%) alloy is used in reconstructive dentistry and cardiovascular implantation like the binary TiNi based alloys with 50.7 and 50.8 at% Ni.

For studying the hydrogen distribution and phase state of Ti_49.4_Ni_50.6_ (at%), the specimens were shaped as plates of dimensions 10 mm × 10 mm and thickness 1.13 mm. Its specimens for studying the average hydrogen concentration were square bars of cross-section 1.1 mm × 1.1 mm and length 50 mm. Both the plates and the bars were mechanically grinded using an abrasive paper with gradual decrease in its grit to 2000 (Automotive Aftermarket 3M United Kingdom PLC, Market Place Bracknell, Berks, UK), polished with a diamond paste grained to 0.6 μm (Pasta Diamanta, Corsico (MI), Italy) and rinsed in ethanol and warm distilled water.

The specimens were hydrogenated at room temperature (295 K) in normal saline (0.9% NaCl aqueous solution with pH = 5.7) at a current density of 20 A/m^2^ for 0.5–6 h. The increase of electrolyte temperature was about 2 K after hydrogenation for 6 h.

The structure and the phase state of Ti_49.4_Ni_50.6_ (at%) were analyzed at 295 K on a DRON-7 diffractometer (CoK_α_ radiation) with PDWin software (JSC IC Burevestnik, St. Petersburg, Russia). It should be noted that in this geometry, the thickness of layers with 95% of the diffraction intensity in CoK_α_ radiation is 2.3–2.9 µm at 2 θ = 20°–25° and 3.5–5.6 µm at 2 θ = 40°–50°, according to estimations proposed elsewhere [32]. The average hydrogen content in Ti_49.4_Ni_50.6_ (at%) was determined on a LECO RHEN 602 gas analyzer (Saint Joseph, MI, USA). The hydrogen distribution in Ti_49.4_Ni_50.6_ (at%) was studied by glow-discharge optical emission spectrometry on a GD OES Profiler 2 (Jobin Yvon Emission Horiba Group, Longjumeau Cedex, France). This method is based on the sputtering of the material of the samples, which are the cathode in the plasma of a glow discharge in argon. The atomized atoms of the sample material are excited and ionized in the glow discharge plasma and emit characteristic radiation. This radiation is analyzed with an optical spectrometer. The sputtering of the sample with argon ions is continuously, layer by layer, as the depth of the erosion crater increases. This makes it possible to obtain profiles of the distribution of elements in the surface layers of the samples by registering the signal of the optical spectrometer depending on the sputtering time. The quality of the analysis depends on the shape of the craters. The craters formed during the sputtering of the Ti_49.4_Ni_50.6_ (at%) samples had vertical walls and a flat bottom, which is optimal. The depth of craters after sputtering for 900 s was 50 ± 3 µm. The average content of hydrogen and its distribution profiles in Ti_49.4_Ni_50.6_ (at%) was determined within 1 h after hydrogenation.

## 3. Results

**Figure 3:**
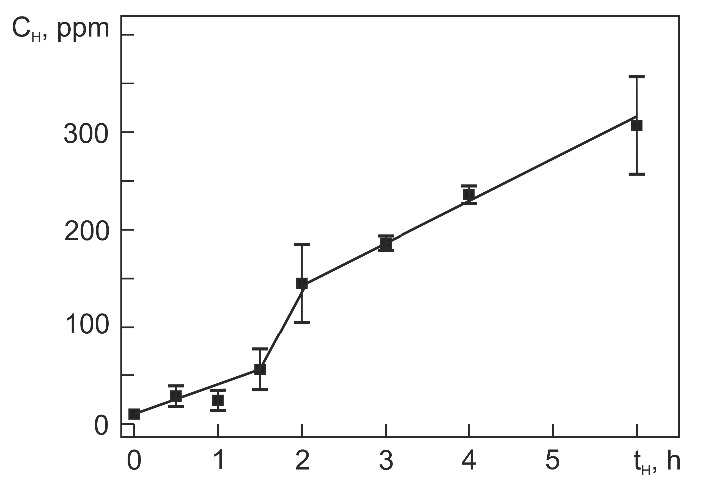
Hydrogen concentration vs. hydrogenation time in Ti_49.4_Ni_50.6_ (at%).

**Figure 4:**
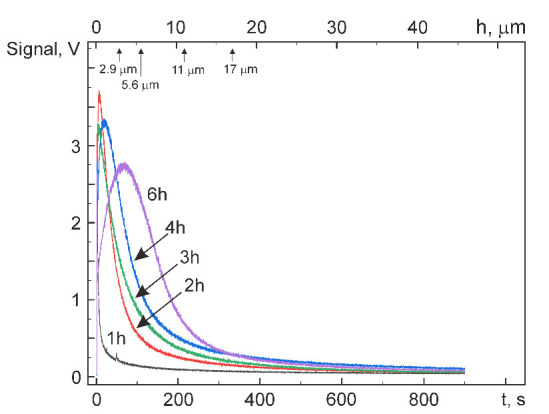
Qualitative profiles of signal distribution due to hydrogen atoms vs. sputtering time in Ti_49.4_Ni_50.6_ (at%) after electrolytic hydrogenation in normal saline. (This figure is in color only in the electronic version.)

Figure 3 shows the average hydrogen concentration C_H_ in Ti_49.4_Ni_50.6_ (at%) versus the hydrogenation time t_H_. It is seen that at up to t_H_ = 1 h, the absorption of hydrogen is slight. The average hydrogen concentration measures 50 wt. ppm (or ppm) even at t_H_ = 1.5 h. After hydrogenation for 1.5 h, its value increases steeply, reaching C_H_ = 150 ppm at t_H_ = 2 h. As the hydrogenation time is increased from 2 to 6 h, the average hydrogen concentration increases almost linearly to C_H_ = 300 ppm. The behavior of C_H_ in Figure 3 characterizes Ti_49.4_Ni_50.6_ (at%) with a uniform hydrogen distribution in its volume. However, directly after hydrogenation, the hydrogen distribution over the alloy cross-section is rather nonuniform. Figure 4 shows the cross-sectional hydrogen distribution versus the hydrogenation time *t*_H_. The profiles in Figure 4 qualitatively characterize the hydrogen distribution because the ordinate is not the absolute concentration of hydrogen but the signal amplitude proportional to its concentration (in the first approximation). The lower abscissa axis is the sputtering time, and the upper one is the depth from the alloy surface calculated for respective time points on the assumption of equal sputtering rates of the hydrogenated layer and initial material far from it.

**Figure 5:**
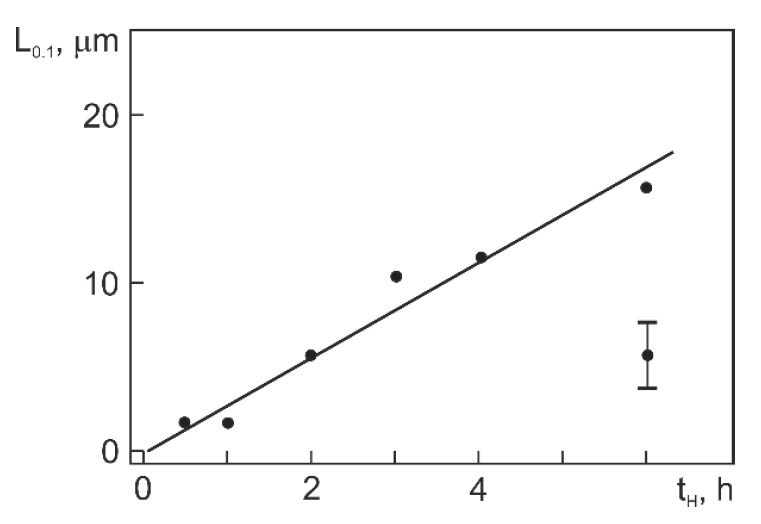
Depth L_0.1_ with hydrogen signal level of 10% from its maximum vs. hydrogenation time in Ti_49.4_Ni_50.6_ (at%).

It is seen from Figure 4 that the localization of hydrogen peaks in Ti_49.4_Ni_50.6_ (at%) falls on its near surface region (3–4 µm thick) and that the highest peak occurs after hydrogenation for 2 h. As t_H_ is increased to 6 h, the signal gets smaller. By and large, the thickness of the layer with high hydrogen content increases monotonically with t_H_ due to hydrogen diffusion deep into the material. Figure 5 shows the depth L_0.1_ at which the signal level measures 10% of its near-surface maximum, allowing us to qualitatively judge the layer thickness with a high hydrogen content compared to its value in the initial alloy (10 ppm). It is seen from Figure 5 that the layer rich in hydrogen almost linearly increases in thickness to ~17 µm as t_H_ is increased to 6 h. Thus, about 90% of hydrogen atoms are concentrated in zones of thickness 11 µm and 17 µm after hydrogenation for 4 and 6 h, respectively. Note that the thickness of these zones is close to the average grain size in a direction normal to the rolling plane (14 µm) coincident with the surface samples. The maximum hydrogen content is in a narrow surface zone (Figure 4) whose width is smaller than L_0.1_ (Figure 5).

**Figure 6:**
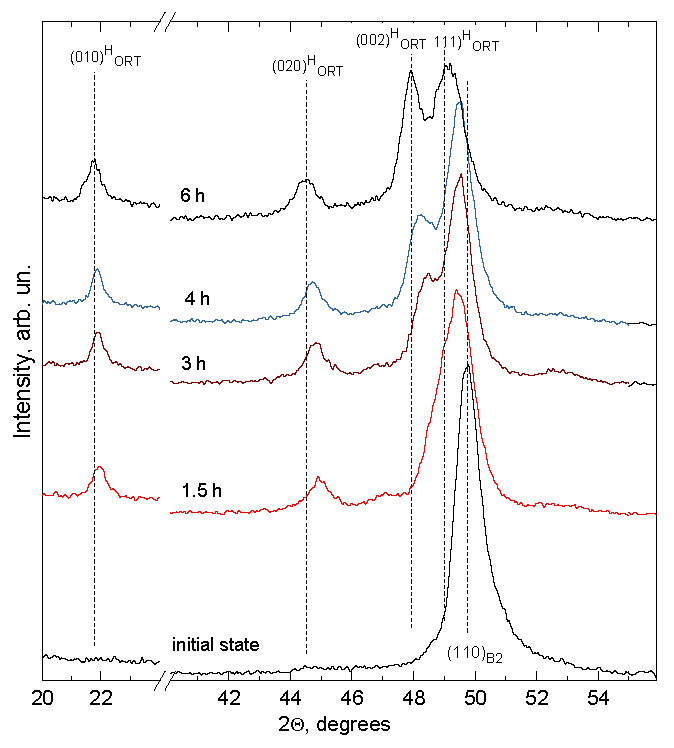
X-ray diffraction patterns of Ti_49.4_Ni_50.6_ (at%) before and after hydrogenation at 295 K in normal saline for 1.5, 3, 4, and 6 h (20 A/m^2^). (This figure is in color only in the electronic version.)

**Figure 7:**
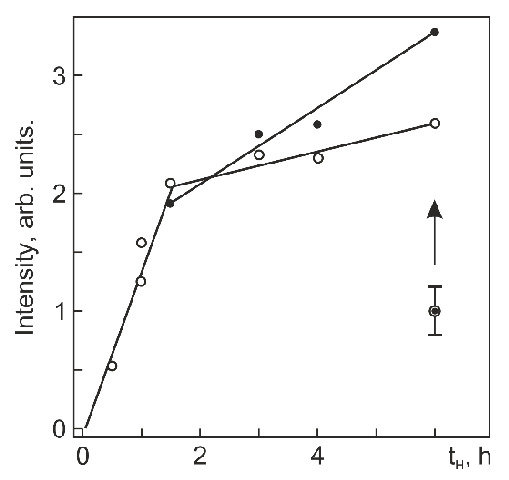
Peak intensity at 2Q ≈ 22° (ο) and 2Q ≈ 45° (●) vs. hydrogenation time.

The surface structure and phase state of Ti_49.4_Ni_50.6_ (at%) after hydrogenation at 295 K for different times can be judged from Figure 6, showing its diffraction patterns in the Bragg–Brentano geometry (θ–2 θ, CoK_α_ radiation). As it was noted above, the thickness of analyzed layers equals 2.3–2.9 μm at 2 θ = 20°–25° and 3.5–5.6 μm at 2 θ = 40°–50°. Thus, the diffraction patterns in Figure 6 reflect the evolution of the structure and phase state of hydrogenated layers whose thickness is close to the surface layer thickness with a maximum hydrogen content and is no greater than ~40% of the average grain size in this direction. It is seen from Figure 6 that before hydrogenation, only a (110)_B2_ peak at 2 θ ≈ 49.7° is detected in the range of diffraction angles 2 θ ≤ 50°. However, even after hydrogenation for 0.5 h, the alloy reveals a weak peak at 2 θ ≈ 22°, and after hydrogenation for 1 and 1.5 h, two peaks at 2 θ ≈ 22° and 2 θ ≈ 45°, respectively, and an increase in the (110)_B2_ asymmetry. After hydrogenation for 3 h and longer, a peak at 2θ ≈ 48° appears. Increasing the hydrogenation time to t_H_ = 6 h shifts the (110)_B2_ peak from 2θ ≈ 49.7° to the range of smaller diffraction angles, suggesting that the corresponding interplanar spacing gets larger. The peak intensity at about 2 θ = 22 ° and 45 ° increases most rapidly with increasing t_H_ to 1.5–2 h, and further, this increase slows down (Figure 7).

Such peak intensities correlate with hydrogen distributions in a surface zone ~5.6 µm thick: after hydrogenation for more than 2 h, hydrogen starts actively diffusing from this zone deep into the alloy but its concentration through a thickness of 2.9–5.6 µm becomes maximal after hydrogenation for 6 h (Figure 4). Thus, the absorption of hydrogen by Ti_49.4_Ni_50.6_ (at%) during electrolytic hydrogenation leads to the formation of the hydride phase in its surface zone measuring no less than 5.6 µm. The structure of this phase is analyzed in detail below.

## 4. Discussion

Of importance for our further analysis is to estimate the hydrogen content in the surface layers of hydrogenated samples (zones with thickness ≤ 5.6 μm). This estimation is based on the average hydrogen concentration C_H_ (Figure 4) and the thickness of layers L_0.1_ in which the content of absorbed hydrogen is high (Figure 5). Let us assume that the total content of hydrogen with its average concentration C_H_ (Figure 5) is uniformly distributed in a layer of thickness L_0.1_. Then, the average hydrogen concentration in this layer can be estimated as follows:

(1) $$C_{H}\left(L_{0.1}\right)=\frac{C_{H}}{4\left[\left(\frac{L_{0.1}}{T}\right)-{\left(\frac{L_{0.1}}{T}\right)}^{2}\right]},$$

where T is the specimen thickness (1.13 mm). In the specimens hydrogenated for 4 and 6 h, C_H_ equal, respectively, to 230 ppm and 300 ppm (1.20 at% and 1.56 at%) and L_0.1_ equal to 11 µm and 17 µm. Then, C_H_(L_0.1_) equal, respectively, ~6000 ppm and ~ 5000 ppm in these specimens. However, the actual hydrogen distribution is nonuniform: the hydrogen content is highest near the surface and decreases in going deep from the surface to L_0.1_. Assuming (in the first approximation) that the hydrogen content decreases linearly through L_0.1_, its maximum near the surface will be about twice higher than C_H_(L_0.1_), i.e., ~12,000 ppm and ~10,000 ppm after hydrogenation for 4 and 6 h, respectively. It is easy to show that the maximum hydrogen content in the surface layer of Ti_49.4_Ni_50.6_ (at%) hydrogenated for 4 and 6 h is ~39.0 at% and ~35.1 at% and that the approximate chemical composition of the layer in the two cases is represented by Ti_30.1_Ni_30.9_H_39.0_ and Ti_32.0_Ni_32.9_H_35.1_ (at%), respectively.

The hydrogen content can be estimated in weight units (ppm) and atomic percent, and also as the ratio of absorbed hydrogen atoms to metal atoms:

(2) $$x=\frac{N_{H}}{N_{\mathrm{Me}}}=\frac{N_{H}}{N_{\mathrm{Ti}}+N_{\mathrm{Ni}}}=\frac{a_{H}}{100-a_{H}},$$

where Me is the number of Ti and Ni atoms and a_H_ is the hydrogen content in atomic percent. Then, the chemical composition of surface zones is represented by TiNiH_0.64_ and TiNiH_0.54_ after hydrogenation for 4 and 6 h, respectively.

**Figure 8:**
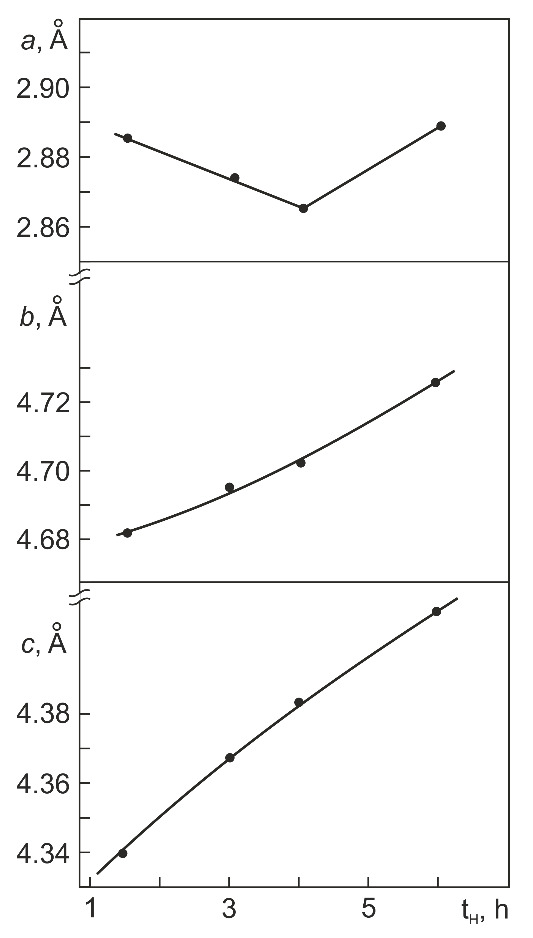
Lattice parameters(a, b and c) of orthorhombic hydride (analogue of β_1_-TiFeH_0.94_) vs. hydrogenation time t_H_ in normal saline at 20 A/m^2^.

It should be noted that the diffraction patterns obtained in our study differ greatly from those typical of TiNi alloys with a monoclinic B19′ and a hydrogen martensite structure (in particular, in interplanar spacing). The identification of TiNiH_x_ on the assumption of its tetragonal structure (with x = 0.30–0.55 [15,22] or x =1 [17,18,19]) is also doubtful because a rather large discrepancy is found between the lattice parameters of tetragonal TiNiH_0.54_ after hydrogenation for 6 h and those of TiNiH_0.55_ [15,22]: *a* = 6.00 Å, *c* = 11.26 Å against *a* = 6.25 Å, *c* = 12.60 Å, respectively. Besides, the volume changes induced by these hydrides of approximate composition differ even in sign: (V_H_–16V_B2_)/16V_B2_ = + 11.7% for TiNiH_0.55_ [15,22] and –4.4% for TiNiH_0.54_ (after hydrogenation in saline for 6 h). Hence, the available data give no way of reliably identifying the hydride structure formed in Ti_49.4_Ni_50.6_ (at%) during electrolytic hydrogenation in normal saline (0.9% NaCl). In this connection, let us extend our analysis to TiMeH_x_ in B2-structured TiMe alloys. According to [33], the hydrides formed in TiFe during saturation in gaseous hydrogen are orthorhombic TiFeH_0.94_ and TiFeH_1.40_ (space group $D_{2h}^{5}$ – Pmcm) with lattice parameters *a* = 2.954 (1) Å, *b* = 4.538 (1) Å, *c* = 4.381 (1) Å and *a* = 3.094 (3) Å, *b* = 4.513 (4) Å, *c* = 4.391 (10) Å, respectively. The most intense reflections of these hydrides are due to their orthorhombic structure: (111), (002), (020), and (010) in order of increasing interplanar spacing. Between the reflections (010) and (020), only (011), (100), and (110) are resolved but their intensity measures 1–2% of the 100% intensity of the strongest reflection (111). The probability of identifying these reflections on X-ray diffraction patterns is low, particularly in view of the coarse-grained structure and texture of rolled specimens. In general, the diffraction patterns of TiFeH_0.94_ and TiFeH_1.40_ presented in the cited paper [33] are qualitatively the same as those suggesting the presence of an orthorhombic hydride phase in Figure 6 with its lattice parameters versus the hydrogenation time in Figure 8.

**Figure 9:**
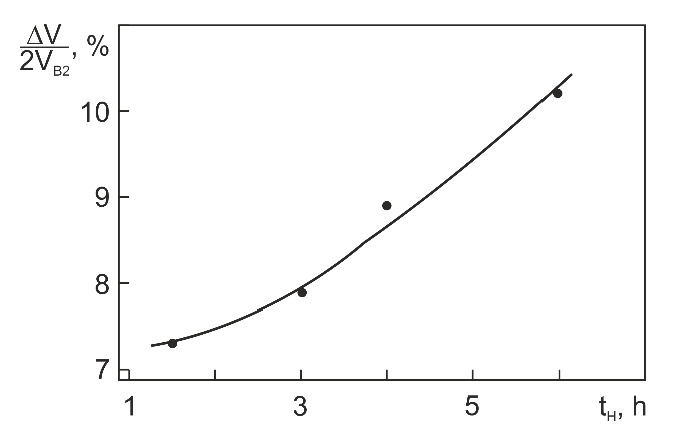
Volume change induced by orthorhombic hydride vs. hydrogenation time.

It is seen in Figure 8 that as the hydrogenation time (and, hence, the amount of absorbed hydrogen) is increased, the lattice parameter *a* first decreases (t_H_ = 1.5–4 h) and then increases (t_H_ > 4 h) while the parameters *b* and *c* grow monotonically with t_H_. The absolute values of the lattice parameters in Figure 8 suggest that the orthorhombic structure of TiNiH_0.54_, like the tetragonal structure of TiNiH_x_ with x = 0.30–0.55, has orientation relations with the initial B2 structure. Behind this assumption is the following reasoning. In Cu-doped TiNi alloys (in particular, in Ti_49.5_Ni_49.5_Cu_10_ (at%) [34]), a B19 martensite phase with a similar orthorhombic structure (space group $D_{2h}^{5}$ – Pmmb) and lattice parameters *a* = 2.881 Å, *b* = 4.279 Å, *c* = 4.514 Å is formed via B2→B19 transformation. The B19 phase has the following orientation relations with the initial B2 phase: [100]_B19_ ‖ [100]_B2_, [010]_B19_ ‖ [01$\bar{1}$]_B2_, [001]_B19_ ‖ [011]_B2_ [34]. During its formation, *a*_B19_ < *a*_B2_ (3.030 Å), *b*_B19_ ≈ *a*_B2_$\sqrt{2}$ (4.285 Å), and *c*_B19_ > *a*_B2_$\sqrt{2}$. On the assumption of similar orientation relations for orthorhombic TiNiH_0.54,_ whose lattice parameters are presented in Figure 8, we can estimate the volume change induced by the formation of this phase. From Figure 9, it is seen that as the hydrogenation time is increased from 1.5 to 6 h, the ratio ∆V/2V_B2_ = (V_H_ − 2V_B2_)/2V_B2_ increases almost linearly from +7.3% to +10.2%. Note that the volume changes induced by the formation of orthorhombic TiNiH_0.54_ (after hydrogenation for 6 h) and tetragonal TiNiH_0.55_ [15,22] are positive and close: +10.2% and +11.3% [15,22], respectively.

Surely, such an orthorhombic hydride structure needs a more detailed confirmation (including its confirmation by transmission electron microscopy). At the same time, our research data suggest that not only a tetragonal hydride, but other hydride structures may arise in TiNi alloys during hydrogenation.

## 5. Conclusions

1. Our study of Ti_49.4_Ni_50.6_ (at%) hydrogenated at 295 K in normal saline at 20 A/m^2^ for 0.5–6 h shows that the average concentration of absorbed hydrogen increases with the hydrogenation time t_H_ as follows: slowly to 50 ppm at t_H_ = 0.5–1.5 h, steeply to 150 ppm at t_H_ = 1.5–2 h, and linearly to 300 ppm at t_H_ = 2–6 h. Increasing the hydrogenation time increases the diffusion of hydrogen deep into the alloy. The hydrogen-rich layer thickness increases linearly with t_H_, measuring ~17 µm at t_H_ = 6 h.
2. The maximum content of absorbed hydrogen in the alloy falls within its surface layer ~5.6 µm thick, which is equal to the layer scanned for diffraction with CoK_α_ radiation in the θ–2 θ geometry at 2 θ ≤ 50°. The composition of this layer corresponds to TiNiH_x_ with x = 0.64 and x = 0.54 (atomic ratio of hydrogen to Ti plus Ni) after hydrogenation for 4 and 6 h, respectively.
3. It is shown that no hydride phase reflections are identified, assuming the presence of the known hydride phase TiNiH_x_ (x from 0.30 to 1.0) with tetragonal structure (space group I4/mmm).
4. It was found that the structure of hydride phase formed in Ti_49.4_Ni_50.6_ (at%) during hydrogenation is similar to the structure of orthorhombic β_1_-TiFeH_0.94_ hydride phase (space group Pmcm). After hydrogenation for 6h, the lattice parameters of TiNiH_0.54_ orthorhombic hydride phase are *a*_H_ = 2.890 Å, *b*_H_ = 4.726 Å, *c*_H_ = 4.410 Å and the volume change induced by it is +10.2%.
